# A patient with single coronary artery, bicuspid aortic valve and sinus of Valsalva aneurysm

**DOI:** 10.1186/s12872-020-01750-4

**Published:** 2021-03-25

**Authors:** Ahmed N. Mohammad, Oghenesuvwe Eboh, Muna Mian, Rony L. Shammas

**Affiliations:** 1 Department of Internal Medicine, East Carolina University, Greenville, NC 27834 USA; 2 Vidant Heart and vascular Care, Vidant Medical Center, Greenville, NC 27834 USA

**Keywords:** Single coronary artery, Sinus of Valsalva aneurysm, Bicuspid aortic valve, Congenital heart defects

## Abstract

**Background:**

We report a rare case of a patient who presented with chest pain and was found to have a constellation of rare cardiac anomalies.

**Case presentation:**

A 67-year-old patient with no past medical history presented with chest pain. He had mild troponin elevation, but no ischemic changes on ECG. He underwent a CT coronary angiogram for further evaluation. He was found to have a type 0 bicuspid aortic valve, large left sinus of Valsalva aneurysm and type R-III single coronary artery. These findings were confirmed with transesophageal echocardiogram and coronary angiogram. He underwent a successful repair of his aortic root aneurysm with a synthetic patch.

**Conclusions:**

The combination of type R-III single coronary artery, bicuspid aortic valve, and left sinus of Valsalva aneurysm congenital anomalies in one individual is extremely rare and marks our case unique. Given the size of his Sinus of Valsalva aneurysm, the patient underwent surgical repair of his aneurysm and was asymptomatic when seen in follow-up.

## Background

Sinus of Valsalva is the aortic root area between the aortic valve annulus and the sinotubular ridge. Normally, it has a diameter of up to 4.0 cm in men and 3.6 cm in women and contains the coronary ostia [1]. The aortic valve normally has three cusps named left coronary, right coronary and non-coronary cusps [2]. The normal coronary artery anatomy is made up of the right and left coronary arteries that arise from their respective ostia. The right coronary artery (RCA) usually emerges from the right cusp and supplies blood to the right atrium, right ventricle, SA node, and AV node. The left main coronary artery (LMCA) normally originates from the left cusp and gives off two major branches, the left anterior descending (LAD) and the left circumflex (LCx) coronary arteries, which supply blood to the left atrium and left ventricle [3, 4]. In about 85% of the population, the RCA is dominant giving both the posterior descending artery (PDA) and the postero-lateral artery (PLA) which supply the inferior part of the interventricular septum and the inferior aspect of the left ventricle, respectively. When both the PDA and PLA are supplied by the Lcx, the circulation can be classified as left dominant (8% of the population). In the remaining 7%, the circulation is co-dominant (PDA from the RCA and PLA from the Lcx) [5]. Here, we present a patient with a constellation of rare anomalies including a large left sinus of Valsalva aneurysm (SOVA), a single coronary artery (SCA) and a bicuspid aortic valve (BAV).

## Case presentation

**Fig. 1:**
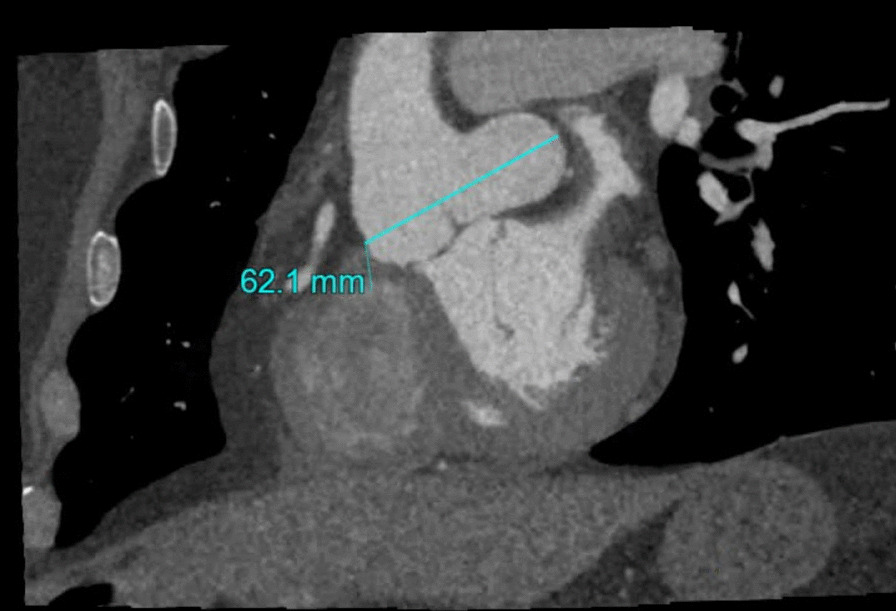
Cardiac CT showing the dilated sinus and the left SOVA

**Fig. 2:**
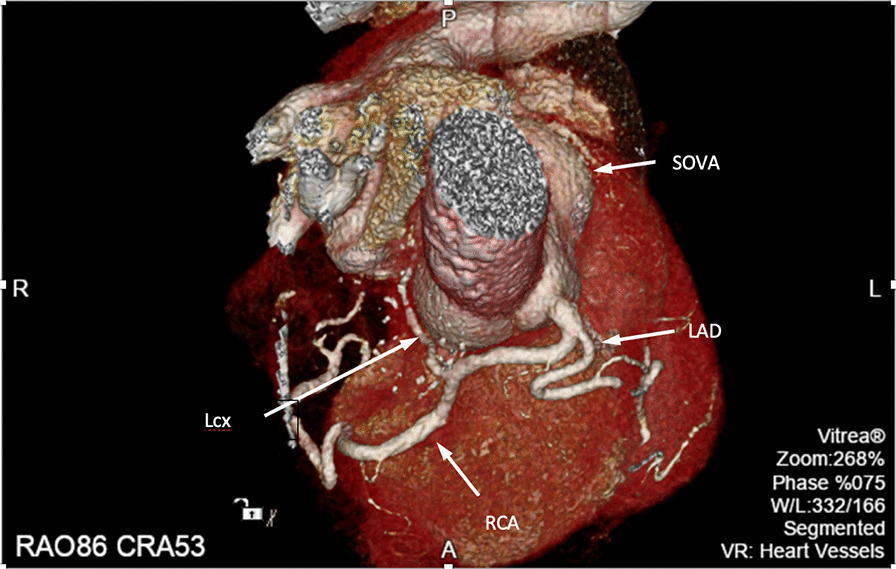
Cardiac CT 3D-rendered images showing the left Sinus of valsalva aneurysm (SOV), the left main origin from the right cusp and left anterior descending artery (LAD) coursing anteriorly while the left circumflex (Lcx) appears to originate from the proximal right coronary artery (RCA) and runs posterior to the aorta. The RCA is seen following its usual course

**Fig. 3:**
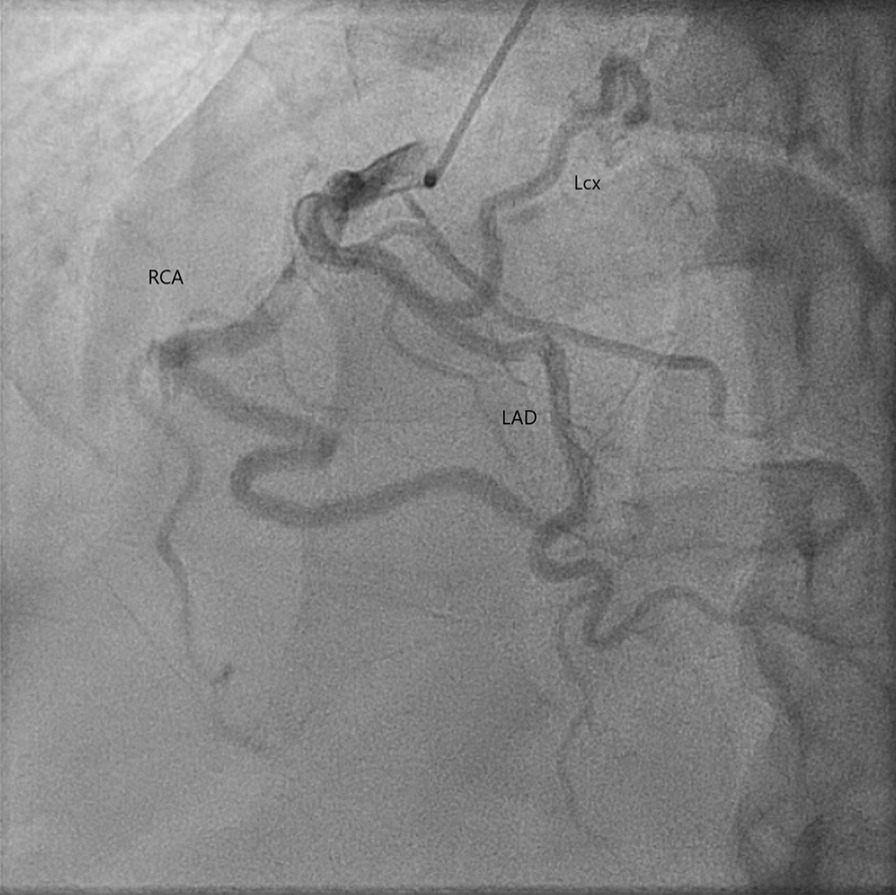
Cardiac catheterization showing the single coronary artery origin from the right cusp with patent vessels

**Fig. 4:**
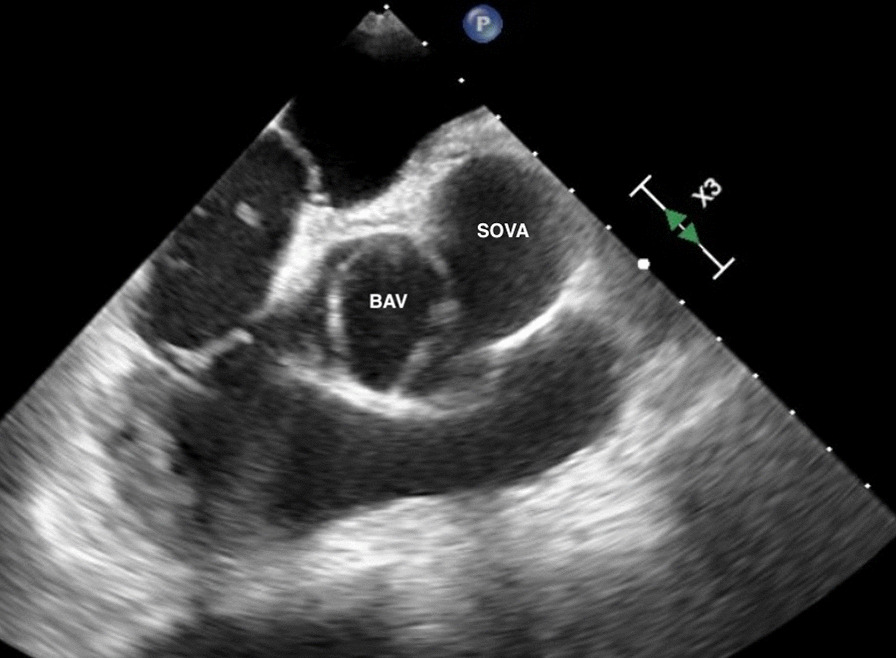
Transesophageal echocardiogram showing the bicuspid aortic valve (BAV) and the left sinus of Valsalva aneurysm(SOVA) adjacent to it

The patient is a 67-year-old African American male with no significant past medical history who presented to the emergency room with chest pain. He described non-radiating, midline, sharp “needlelike” chest pain that occured during activity and was associated with shortness of breath. It was 8/10 in severity and lasted for less than a minute and subsided with rest. Electrocardiogram revealed left ventricular hypertrophy with incomplete right bundle branch block and left anterior fascicular block. He had borderline troponin I elevation of 0.04 ng/ml (Reference range ≤ 0.03 ng/ml). Transthoracic echocardiogram was of poor quality but showed normal ejection fraction with no wall motion abnormalities. He remained chest pain free at the hospital and underwent a nuclear stress test which showed a small area of mild reversible perfusion defect in the apical left ventricular segment. Due to the equivocal nature of the myocardial perfusion scan, we proceeded with a CT coronary angiogram for further assessment. He was found to have a type 0 (no raphe) BAV with aortic root aneurysmal dilatation due to a large left SOVA measuring 6.2 × 4.4 cm (Figs. 1, 2). Interestingly, he was also noted to have a SCA that arose from the anterior right coronary cusp (Fig. 2). His LAD crossed anteriorly over the right ventricular outflow tract (RVOT) and the LCx coursed posteriorly behind the aorta. No obstructive coronary artery disease was found. These findings were confirmed on coronary angiogram which was requested prior to surgical repair of his SOVA (Fig. 3). Transesophageal Echocardiogram confirmed presence of bicuspid aortic valve and large left SOVA (Fig. 4). There was no aortic stenosis or regurgitation. Patient underwent a successful SOVA repair with synthetic patch.

## Discussion and conclusions

Coronary artery anomalies are rare and include anomalies of origin, distribution and fistulae. Although many are benign, some cases which involve an aberrant coronary artery crossing in between the pulmonary artery and the aorta are considered malignant and may lead to sudden cardiac death. This is especially true for cases in which ischemia or symptoms related to the anomaly are present. In such cases surgery is indicated (Class 1). Even in the absence of symptoms or ischemia, surgery is reasonable (Class 2A) for anomalous origin of the left coronary artery from the right sinus [6]. SCA is a congenital anomaly defined as a solitary coronary artery arising from a single coronary ostium and has a prevalence of 0.024 to 0.066% [7]. Lipton et al. described several subtypes based on angiographic findings. The classification uses letters and roman numerals depending on the ostial origin of the SCA (R type for right ostium, L type for left ostium) and the anatomy of its distribution (I, II, or III). Furthermore, the letters A, B, P, (anterior, between and posterior respectively) are used to outline the course of the vessel in relation to the pulmonary artery and the aorta [8].

We report a rare case of SCA, BAV and large left SOVA. The SCA originated from the anterior right coronary cusp and gave rise to a large dominant RCA which followed a normal course and LAD that crossed anterior to pulmonary artery. The small circumflex artery originated from the proximal RCA and coursed posterior to the ascending aorta. Our patient falls into the R-III category Per Lipton et al. classification. Lipton type R-III is one of the least common SCA anomalies with an incidence rate of 0.004% [9]. Our patient did not have a malignant coronary anatomy as his LAD crossed anterior to the pulmonary artery and right ventricular outflow tract (RVOT) and his LCx coursed posterior to the aorta.

Moreover, our patient was found to have bicuspid aortic valve and left SOVA. The association between SCA and bicuspid aortic valve is rare, however, has been described before in the literature [10, 11]. SOVA has also been infrequently reported with BAV [12]. Isolated bicuspid aortic valve is the most common congenital heart defect with an estimated prevalence of 1–2% [13]. SOVA can be congenital or acquired with an incidence of < 0.1% in the general population. It usually affects the right coronary sinus with left SOVA involvement being least common [14]. Most cases of SOVA are discovered incidentally. Serious complications are usually related to aneurysmal rupture.

In our case, the patient's troponin elevation was borderline, flat and not diagnostic for acute coronary syndrome. His chest pain was atypical in quality (“needlelike”), although was related to exertion. Based on the quality of chest pain, absence of objective findings and results of imaging studies it is likely that his presenting chest pain was non cardiac. Another, though less likely explanation, is presence of microvascular disease. The combination of R-III SCA, BAV, and left SOVA congenital anomalies in one individual is extremely rare and marks our case unique. It remains unclear whether his chest pain on presentation was related to any of these findings; however, given the size of his Sinus of Valsalva aneurysm, the patient underwent SOVA repair. He was seen in follow up after 4 weeks and was doing well with no symptoms. He will continue to undergo periodic follow-up and monitoring for his BAV.

## Data Availability

The data are available from the corresponding author on reasonable request and with permission of Vidant medical center.

## Abbreviations

SCA: Single coronary artery
SOVA: Sinus of Valsalva aneurysm
BAV: Bicuspid aortic valve
RCA: Right coronary artery
LAD: Left anterior descending
LMCA: Left main coronary artery
LCx: Left circumflex
PDA: Posterior descending artery
PLA: Postero-lateral artery
RVOT: Right ventricular outflow tract
